# Alternations of Circadian Clock Genes Expression and Oscillation in Obstructive Sleep Apnea

**DOI:** 10.3390/jcm8101634

**Published:** 2019-10-06

**Authors:** Ming-Yu Yang, Pei-Wen Lin, Hsin-Ching Lin, Pai-Mei Lin, I-Ya Chen, Michael Friedman, Chi-Fa Hung, Anna M. Salapatas, Meng-Chih Lin, Sheng-Fung Lin

**Affiliations:** 1 Department of Otolaryngology, Kaohsiung Chang Gung Memorial Hospital and Chang Gung University College of Medicine, Kaohsiung 83301, Taiwan; yangmy@gmail.com; 2 Graduate Institute of Clinical Medical Sciences, College of Medicine, Chang Gung University, Tao-Yuan 33302, Taiwan; jupiter670516@yahoo.com.tw; 3 Division of Glaucoma, Department of Ophthalmology, Kaohsiung Chang Gung Memorial Hospital and Chang Gung University College of Medicine, Kaohsiung 83301, Taiwan; lpw324@adm.cgmh.org.tw; 4 Sleep Center, Kaohsiung Chang Gung Memorial Hospital and Chang Gung University College of Medicine, Kaohsiung 83301, Taiwan; mengchih@cgmh.org.tw; 5 Robotic Surgery Center, Kaohsiung Chang Gung Memorial Hospital and Chang Gung University College of Medicine, Kaohsiung 83301, Taiwan; 6 Department of Nursing, I-Shou University, Kaohsiung 84001, Taiwan; paimei@isu.edu.tw; 7 Department of Otolaryngology—Head and Neck Surgery, Division of Sleep Surgery, Rush University Medical Center, Chicago, IL 60612, USA; mfriedman@chicagoent.com; 8 Department of Otolaryngology, Advanced Center for Specialty Care, Advocate Illinois Masonic Medical Center, Chicago, IL 60657, USA; anna.salapatas@gmail.com; 9 Department of Psychiatry, Kaohsiung Chang Gung Memorial Hospital and Chang Gung University College of Medicine, Kaohsiung 83301, Taiwan; chifa.hung@gmail.com; 10 Division of Pulmonary and Critical Care Medicine, Department of Internal Medicine, Kaohsiung Chang Gung Memorial Hospital and Chang Gung University College of Medicine, Kaohsiung 83301, Taiwan; 11 Division of Hematology and Oncology, Department of Internal Medicine, E-Da Hospital, Kaohsiung 84001, Taiwan; shlintw@yahoo.com.tw

**Keywords:** obstructive sleep apnea/hypopnea (OSA), circadian rhythm, circadian clock genes

## Abstract

Circadian misalignment plays an important role in disease processes and can affect disease severity, treatment outcomes, and even survivorship. In this study, we aim to investigate whether expression and daily oscillation patterns of core circadian clock genes were disturbed in patients with obstructive sleep apnea/hypopnea (OSA) syndrome. We performed real-time quantitative reverse transcriptase-polymerase chain reactions to examine the expression of the nine core circadian clock genes in leukocytes of peripheral blood collected at 12 AM, 6 AM, 12 PM, and 6 PM from 133 patients with OSA and 11 normal controls. Daily expression patterns of the nine circadian clock genes were observed in normal controls, but three of these genes (*BMAL1, CLOCK, CRY2*) were disrupted in patients with OSA. The expressions of eight circadian clock genes (except *PER1*) at midnight were significantly downregulated in patients with severe OSA. Binary logistic regression analysis selected *CRY1* and *PER3* as independent factors for severe OSA and showed that the combined expressions of *CRY1* and *PER3* enhanced the capability of predicting severe OSA (Odds ratio, 5.800; 95% CI, 1.978 to 17.004; *p* = 0.001). Our results show that combined expressions of *CRY1* and *PER3* at midnight could be a potential predictor for severe OSA.

## 1. Introduction

Obstructive sleep apnea/hypopnea (OSA) syndrome is characterized by repetitive obstruction of the upper airway and causes repetitive hypoxia/reoxygenation. In untreated OSA patients, this ultimately results in an increased risk of hypertension, coronary artery disease, myocardial infarction, stroke, and even sudden death [1,2,3,4,5].

“Circadian clock” means an internal time keeping system that developed from organisms which allows them to anticipate one of the most profound environmental signals, the daily cycle of light/dark. In mammals, circadian rhythm refers to the “body clock” which is an endogenously driven, nearly 24-hour cycle in biochemistry, physiology, or behavior, such as sleep and activity, appetite, hormone levels, metabolism, and gene expression [6]. The circadian master clock resides in the suprachiasmatic nucleus in the hypothalamus. It is now accepted that peripheral blood (PB) cells contain a circadian clock similar to that in the suprachiasmatic nucleus [7]. Until now, at least nine mammalian core circadian clock genes have already been identified: *Period1* (*PER1*), *Period2* (*PER2*), *Period3* (*PER3*), *CLOCK*, *Cryptochrome1* (*CRY1*), *Cryptochrome2* (*CRY2*), *BMAL1*, *Casein kinase 1ε* (*CK1ε*), and *Timeless* (*TIM*). The regulation relies on positive loops (such as *CLOCK* and *BMAL1*) and negative loops (such as *PER1*, *PER2*, *PER3*, *CRY1*, *CRY2* and *TIM*) in the oscillators [8]. Circadian rhythms are ubiquitous phenomena, and are found, for example, in the sleep-wake cycle, body-core temperature, leukocyte count, trace metal concentrations, and levels of many hormones, such as cortisol and melatonin [9,10]. A stable human biological clock helps to regulate sleep patterns, hormone release, and blood pressure, etc. Many aspects of cardiovascular or cerebrovascular physiology are subject to diurnal variation. Serious adverse events including cancers, atherosclerosis, myocardial infarction, and stroke are significantly associated with the disturbance of circadian gene expressions [9].

Until now, there have been very limited reports on the relationships between circadian clock genes and OSA. Sleep is a neurological generated phenomenon who’s timing is under circadian influences. Sleep–wake cycle could modulate circadian clock genes [11] and in turn circadian system could influence apnea occurrence and duration [12,13]. Therefore, in this study, we prospectively investigated the expression and daily oscillation patterns of the nine core circadian clock genes in patients with OSA to further understand the impact of circadian clock regulation on OSA.

## 2. Materials and Methods

### 2.1. Patients, Normal Controls, and Samples

The design of this study adhered to the tenets of the Declaration of Helsinki. This study was approved by the Institutional Review Board (IRB) of the Chang Gung Memorial Hospital (CGMH) Ethical Committee (CGMH IRB No. 102-6026B). Written informed consent was obtained from all participants.

We enrolled 144 subjects with the symptoms of sleep-related breathing disorders such as loud snoring, observed apnea or excessive daytime sleepiness, from our sleep clinics. All of the 144 subjects were consecutively admitted for a full-night polysomnography (PSG) at the sleep center of Kaohsiung Chang Gung Memorial Hospital (KCGMH), a 2,600-bed tertiary referral medical center. The criteria for diagnosing OSA are as previously described [14,15,16]. Among the 144 participants, 133 patients were diagnosed with OSA (aged 21–67 years) and 11 subjects were diagnosed as without OSA (aged 26–59 years), and these 11 subjects were categorized as normal controls. The subjects enrolled in this study did not experience shift work or jet lag 1 week before the experiment. Female individuals included in this study were not in their menstrual phases during the experiment. The time schedule and the daily activity of normal controls and patients with OSA were not restricted but they were asked to have breakfast between 7:00 and 8:00, lunch between 11:30 and 13:30, and dinner between 17:30 and 19:30; they were also asked to go to sleep before 24:00 and wake up at around 7:00. Before the PSG, all participants were asked to follow the pre-arranged schedule as described above strictly for 1 week. The collection of PB was carried out four times daily at 12 AM (00:00), 06 AM (06:00), 12 PM (12:00), and 06 PM (18:00), respectively, at the sleep center of KCGMH. For the PB collection at 12 AM, blood-drawing was done under minimal (~20 lux) light density and all individuals fell asleep immediately after blood was drawn.

### 2.2. Analysis of Expression of Circadian Clock Genes

Isolation of total PB leukocytes, RNA extraction and cDNA synthesis were performed as previously described [17,18]. The expression of the nine circadian clock genes and *ACTB* gene (as endogenous reference control) was analyzed using real-time quantitative reverse transcriptase- polymerase chain reaction (qRT-PCR) as previously described [17,18]. The relative gene expression was calculated by equalizing differences to the Δ*C_t_* of 6 PM in normal controls (i.e., 2^−(^^Δ*Ct* of X -^^Δ*Ct* of 6PM)^); values were normalized so that the expression level of normal controls at 6 PM equaled 1.0.

### 2.3. Statistical Analysis

Student’s *t*-test was used to detect the differences of clinical parameters between groups of normal controls and OSA and among groups of normal controls and different severities of OSA. Repeated-measures ANOVA (Analysis of variance) was used to detect the differences among four different time points in the expression of each circadian clock gene. The comparison of daily patterns between different groups of individuals in the expression of each circadian clock gene and the comparison of gene expression of the same gene at the same time point between different groups were evaluated with Univariate analysis of General Linear Model (GLM) with post hoc Bonferroni comparison under GLM. Receiver Operating Characteristic (ROC) curve was plotted and ROC area under the curve (AUC) was calculated to compare the discriminating ability of circadian clock genes. Binary logistic regression modeled the effects of selected independent variables on whether or not the expression of a specific gene could be a predictive marker for severe OSA. The values of Δ*Ct* were used for all statistical analysis. All tests were two-sided with statistical significance set at 0.05 and all computations were made using SPSS 22.0 software (IBM SPSS Statistics for Windows, IBM, Armonk, NY, USA) and Graph Pad Prism 7.04 (GraphPad, San Diego, CA, USA).

## 3. Results

### 3.1. Categorization of Patients with OSA

The severities of 133 patients with OSA enrolled in this study were categorized into three major groups according to their apnea/hypopnea index (AHI, /hr.): (1) mild, 5 ≤ AHI < 15, *n* = 27; (2) moderate, 15 ≤ AHI < 30, *n* = 27; and (3) severe, AHI ≥ 30 (*n* = 79); their clinical characteristics are listed in Table 1.

### 3.2. Circadian Patterns of Expression of Circadian Clock Genes in Healthy Controls and Abolished Daily Patterns in Patients with OSA

**Figure 1:**
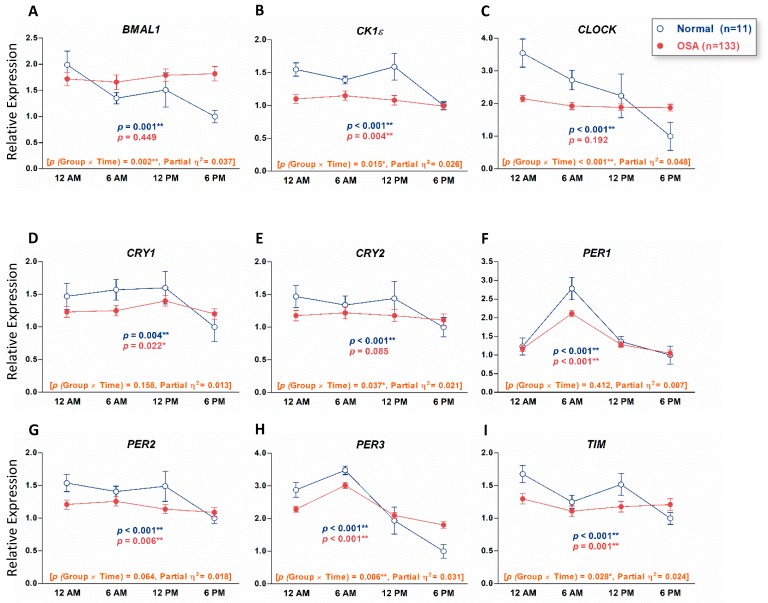
Circadian patterns of the nine circadian clock genes in human peripheral blood (PB) of 133 patients with obstructive sleep apnea (OSA) and 11 normal controls (individuals without OSA). The nine circadian clock genes are *BMAL1* (**A**), *CK1ε* (**B**), *CLOCK* (**C**), *CRY1* (**D**), *CRY2* (**E**), *PER1* (**F**), *PER2* (**G**), *PER3* (**H**) and *TIM* (**I**). The x-axis indicates the time points that PB samples were collected. The y-axis represents the relative mRNA expression level. The value of the mRNA expression at 6 PM in normal controls is designated 1, and the levels of all other mRNA expressions are calibrated to this value. The *p* values indicated were evaluated with Repeated-measures ANOVA. The numbers in brackets are *p* values of group × time interaction and partial η2, respectively. ** *p* < 0.01, * *p* < 0.05.

**Figure 2:**
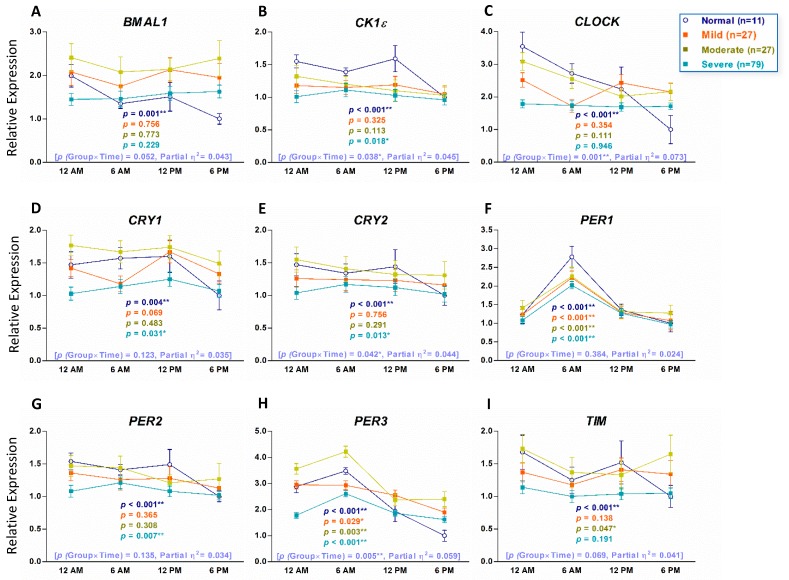
Circadian patterns of the nine circadian clock genes in human peripheral blood (PB) of 133 patients with different severities of obstructive sleep apnea (OSA) and 11 normal controls. The nine circadian clock genes are *BMAL1* (**A**), *CK1ε* (**B**), *CLOCK* (**C**), *CRY1* (**D**), *CRY2* (**E**), *PER1* (**F**), *PER2* (**G**), *PER3* (**H**) and *TIM* (**I**). The *x*-axis indicates the time points that PB samples were collected. The *y*-axis represents the relative mRNA expression level. The value of the mRNA expression at 6 PM in normal controls is designated 1, and the levels of all other mRNA expressions are calibrated to this value. The *p* values indicated were evaluated with Repeated-measures ANOVA. The numbers in brackets are *p* values of group x time interaction and partial η^2^, respectively. ** *p* < 0.01, * *p* < 0.05.

To examine if the expression of circadian clock genes in healthy individuals without OSA showed a time-dependent variation, we used qRT-PCR to analyze PB total leukocytes from 11 healthy individuals who were evaluated by PSG and diagnosed without OSA as normal controls for this study. Repeated-measures ANOVA analyses indicated that in normal controls, all the nine circadian clock genes displayed a time-dependent variation pattern (Figure 1). We also analyzed PB total leukocytes from 133 patients with OSA to examine whether their daily patterns were different from those of normal controls. Repeated-measures ANOVA analyses showed the daily patterns of the *BMAL1*, *CLOCK*, and *CRY2* genes that changed over time in normal controls and were abolished in patients with OSA (Figure 1A,C,E). We further divided patients with OSA into mild, moderate, and severe groups and analyzed their daily patterns of expression of circadian clock genes. Repeated-measures ANOVA analyses showed that the daily patterns of *PER1* and *PER3* were consistent in normal controls and all three groups of patients with OSA (Figure 2F,H). Transcripts of *BMAL1* and *CLOCK* showed time variation in normal controls but the daily patterns were abolished in all three groups of patients with OSA (Figure 2A,C). Transcripts of *CK1ε*, *CRY1*, *CRY2*, and *PER2* that changed over time in normal controls were abolished in mild and moderate groups of patients with OSA and the time-dependent variation recovered in severe groups of patients with OSA (Figure 2B,D,E,G). The comparison between recovered oscillation patterns of *Ck1ε* (*p* = 0.069), *CRY1* (*p* = 0.316), *PER1* (*p* = 0.334), and *PER2* (*p* = 0.122) and their normal control was evaluated by Repeated-measures ANOVA.

### 3.3. Downregulation of Circadian Clock Genes in Patients with OSA at Midnight

**Figure 3:**
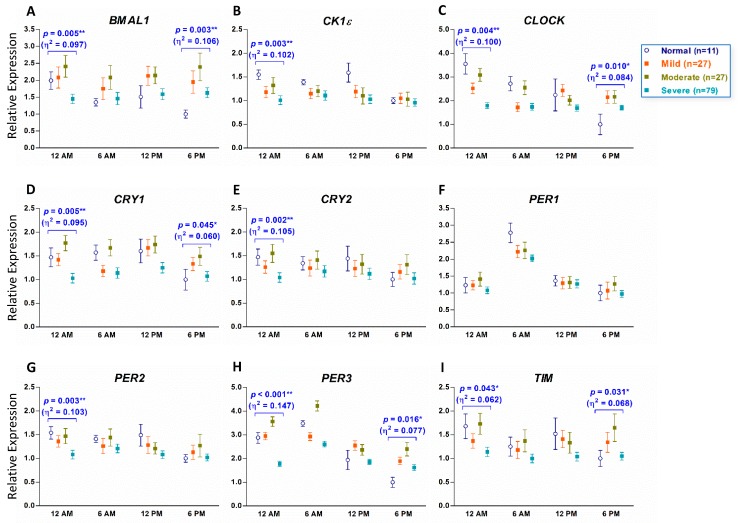
Expression of the nine circadian clock genes at different time points in human peripheral blood (PB) of patients with obstructive sleep apnea (OSA) and individuals without OSA. The nine circadian clock genes are *BMAL1* (**A**), *CK1ε* (**B**), *CLOCK* (**C**), *CRY1* (**D**), *CRY2* (**E**), *PER1* (**F**), *PER2* (**G**), *PER3* (**H**) and *TIM* (**I**). The x-axis indicates the time points that PB samples were collected. The y-axis represents the relative mRNA expression level. The value of the mRNA expression at 6 PM in normal controls (individuals without OSA) is designated 1, and the levels of all other mRNA expressions are calibrated to this value. The *p* values indicated were evaluated with a Univariate analysis of General Linear Model (GLM) with post hoc Bonferroni comparison under GLM. ** *p* < 0.01, * *p* < 0.05. The number in the parentheses is the value of partial η^2^ evaluated with Repeated-measures ANOVA.

Transcripts of *BMAL1* (*p* = 0.005, η^2^ = 0.097), *CK1ε* (*p* = 0.003, η^2^ = 0.102), *CLOCK* (*p* = 0.004, η^2^ = 0.100), *CRY1* (*p* = 0.005, η^2^ = 0.095), *CRY2* (*p* = 0.002, η^2^ = 0.105), *PER2* (*p* = 0.003, η^2^ = 0.103), *PER3* (*p* < 0.001, η^2^ = 0.147), and *TIM* (*p* = 0.043, η^2^ = 0.062) were significantly downregulated at 12 AM in the severe group of patients with OSA (Figure 3A–E,G–I). *PER1* is the only gene that its expression was not different among normal controls and all three groups of patients with OSA at four different time points (Figure 3F). At 6 PM, the expression levels of *BMAL1* (*p* = 0.003, η^2^ = 0.106), *CLOCK* (*p* = 0.010, η^2^ = 0.084), *CRY1* (*p* = 0.045, η^2^ = 0.060), *PER3* (*p* = 0.016, η^2^ = 0.077), and *TIM* (*p* = 0.031, η^2^ = 0.068) were slightly different among the four groups (Figure 3A,C,I).

### 3.4. Correlation and Regression Analysis of Expression of Circadian Clock Genes at Midnight in Patients with OSA

**Figure 4:**
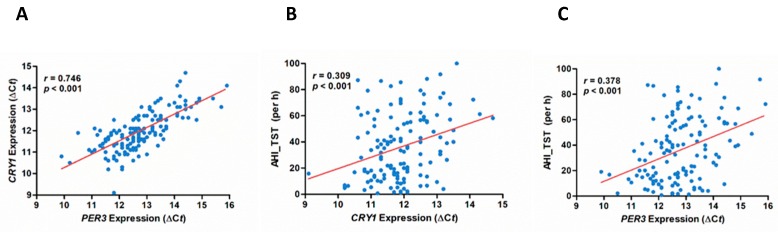
Correlations of expression of *CRY1* and *PER3* with apnea/hypopnea index of total sleep time (AHI_TST). Correlation between (**A**) expression of *CRY1* and *PER3*, (**B**) expression of *CRY1* and AHI_TST, and (**C**) expression of *PER3* and AHI_TST. The values of Δ*Ct* of *CRY1* and *PER3* obtained from real-time quantitative polymerase chain reaction were used for the expression of *PER3* and *CRY1* in Pearson’s correlation with AHI_TST (per hour). The *r* values indicated are the Pearson’s correlation coefficients. Both *r* and *p* values indicated were evaluated with Pearson’s correlation test.

We constructed ROC curves and calculated the AUC to examine the discriminative performance of expression of circadian clock genes at 12 AM between the severe OSA group and non-severe OSA groups. Binary logistic regression analysis selected *CRY1* (Odds ratio, 2.963; 95% CI, 1.277 to 6.879; *p* = 0.011) and *PER3* (Odds ratio, 3.746; 95% CI, 1.595 to 8.795; *p* = 0.002) as independent factors for severe OSA (Table 2). The combined expression of *CRY1* and *PER3* enhanced the prediction of severe OSA (Odds ratio, 5.800; 95% CI, 1.978 to 17.004; *p* = 0.001). Pearson correlation analysis showed significant correlations between CRY1 expression and AHI (r = 0.378, *p* < 0.001) (Figure 4B) and between *PER3* expression and AHI (r = 0.309, *p* < 0.001) (Figure 4C). Our results demonstrated that when the expression of either *CRY1* or *PER3* is low at midnight, the risk to develop severe OSA is about 3- to 4-fold higher than normal. When the expression of *CRY1* and *PER3* are both low at midnight, the risk to develop severe OSA is 5.8-fold higher than normal. Thus, the combined expression of *CRY1* and *PER3* at midnight might have potential to be a predictor for patients with severe OSA.

## 4. Discussion

In this current study, we investigated the nine comprehensive circadian clock genes in patients with OSA and found that the transcripts of all nine circadian clock genes displayed daily patterns in PB of normal controls but three of them, *BMAL1*, *CLOCK* and *CRY2*, were arrhythmic in patients with OSA. Burioka et al. examined the *PER1* gene only and demonstrated that the expression of *PER1* gene peaked at 6AM both in patients with OSA and healthy individuals [19], which coincided with our findings. Our results of *BMAL1, CK1ε*, *CRY1*, *CRY2, PER1*, *PER2,* and *PER3* in normal controls are also consistent with previous reports [18,20,21,22,23,24], demonstrating that the expression of these genes displayed daily oscillation patterns. However, in contrast to our results, previous reports demonstrated that the expression of *CLOCK* was arrhythmic in human PB [18,22]. Since all the normal controls enrolled in this study are examined by a full-night PSG and diagnosed as without OSA, this may explain the inconsistency between our study and other studies that used normal controls with different inclusion criteria or just simply mentioned the healthy subjects, without confirming via a sleep study.

Consistent with our current study, the expression of clock genes has been shown to be altered in patients with OSA [19,25]. Moreira et al. further revealed that the altered expression of *CLOCK* gene in seven patients with OSA was not reverted by continuous positive airway pressure (CPAP) treatment [25]. In contrast, Burioka et al. demonstrated that the *PER1* expression elevated at 02:00 in patients with OSA was significantly decreased by CPAP treatment [19]. In our study, although we observed that the daily oscillation patterns of circadian clock genes were altered in OSA, the changes of daily oscillation patterns seem not as strongly related to the severities of OSA as the expression levels at midnight. We found that the expression levels of *CRY1* and *PER3* were significantly decreased at midnight and might potentially predict severe OSA. A recent study also demonstrated a decrease of *PER3* in veterans with sleep apnea [26]. *PER3* was thought to be less important in the *PERIOD* gene family but more and more human studies have suggested that *PER3* may also be essential in maintaining circadian rhythm [20,27,28,29]. 

The limitations of this study are the small number of normal controls and only four time points of PB were collected. For better comparison for OSA, we used more strict criteria for selecting normal controls, and only individuals examined by PSG and diagnosed without OSA were enrolled. Therefore, only limited numbers of normal controls were qualified. Our concern is that the normal expression and oscillation patterns of circadian clock genes may not be fully reflected by the limited case numbers as the partial η^2^ values in Figure 1 and Figure 2 are small (0.01 ≤ η^2^ ≤ 0.06). Besides, it was very difficult to collect PB from patients more than four times a day although more time points will more precisely reflect the oscillation patterns of circadian clock genes. Although we demonstrated that the expression of *CRY1* and *PER3* genes at 12AM might be able to predict the OSA disease severity, especially for severe OSA, we only revealed the relationship between circadian clock genes and OSA instead of causal effects. In severe OSA, the expression of *CRY1* and *PER3* (*r* = 0.746, *p* < 0.001) at midnight was highly correlated (Figure 4A) but the correlation between *CRY1* and AHI (*r* = 0.309, *p* < 0.001) and between *PER3* and AHI (*r* = 0.378, *p* < 0.001) was not very high. If changes of expression of circadian clock genes could be applied for predicting treatment outcome of OSA, further studies will be needed.

The results of this study may not be sufficient for clinical diagnosis of OSA at present but they do provide a new reference for evaluating the severities of OSA and new directions for future studies. We believe the analysis of expression of circadian clock genes of post-operational OSA patients will strengthen our conclusion. In addition, investigating the mechanisms of downregulated *CRY1* and *PER3* in severe OSA and the molecular causal effects of circadian clock genes and OSA will also let us take a step forward to the understanding of OSA.

## 5. Conclusions

This study provides a comprehensive circadian genes survey for patients with OSA. Our results show that the combined expression of *PER3* and *CRY1* circadian genes at midnight might be a potential predictor for patients with OSA, especially for those with severe OSA. This implementable genetic tool may help clinicians to identify patients with OSA. 

## Figures and Tables

**Table 1:** Clinical characteristics of patients with obstructive sleep apnea/hypopnea (OSA) syndrome.

Characteristic	Normal Controls (*n* = 11)	OSA (*n* = 133)	*p*-Value (1) ^a^	Mild (*n* = 27)	Moderate (*n* = 27)	Severe (*n* = 79)	*p*-Value (2) ^b^
Sex, Male/Female	5/6	111/22	0.002	19/8	24/3	68/11	<0.005
Age, year	42.55 ± 3.45 ^c^	42.47 ± 0.91	0.981	40.89 ± 2.16	44.15 ± 2.23	42.43 ± 1.12	0.736
BMI, kg/m^2^	23.33 ± 0.87	26.08 ± 0.28	0.007	24.39 ± 0.55	24.24 ± 0.42	27.29 ± 0.35	<0.001
ESS	7.6 ± 1.3	8.98 ± 0.38	0.333	8.8 ± 0.9	8.7 ± 0.8	9.2 ± 0.5	0.739
ESS, no. ≤10/>10	7/4	85/48	1.000	19/8	18/9	48/31	0.821
AHI, /hour	2.78 ± 0.44	39.94 ± 2.14	<0.001	10.3 ± 0.58	20.80 ± 0.79	56.59 ± 2.00	<0.001
AHI in REM, /hour	4.58 ± 1.14	42.71 ± 2.01	<0.001	19.55 ± 3.22	34.02 ± 322	53.88 ± 2019	<0.001
% of O_2_ < 90%, %	0.22 ± 0.15	9.85 ± 1.16	0.018	1.20 ± 0.31	3.09 ± 0.64	15.19 ± 1.70	<0.001
Average O_2,_ %	96.96 ± 0.20	95.02 ± 0.21	0.008	96.53 ± 0.18	96.13 ± 0.23	94.12 ± 0.29	<0.001
LSAT, %	90.64 ± 0.69	75.60 ± 1.13	<0.001	86.11 ± 0.92	81.19 ± 1.85	70.10 ± 1.45	<0.001

^a^*p* value (1) is the *p* value for comparison between Normal Controls and OSA. The *p* values indicated were evaluated with Student *t*-test, except for sex (Chi-square). ^b^ *p* value (2) is the *p* value for comparison among Normal Controls and different severities of OSA. ^c^ Data are presented as mean ± standard error of mean. OSA, obstructive sleep apnea/hypopnea; BMI, body mass index; ESS, Epworth sleepiness scale; AHI, apnea/hypopnea index; REM, rapid eye movement; LSAT, lowest saturation of O_2_.

**Table 2:** Binary logistic-regression analysis of predictors of severe obstructive sleep apnea/hypopnea syndrome by expression of *CRY1* and *PER3*.

Independent Factor	Odds Ratio (95% CI)	*p* Value
*CRY1*	2.963 (1.277–6.879)	0.011
*PER3*	3.746 (1.595–8.795)	0.002
None	Reference ^a^	
*CRY1* or *PER3*	2.363 (0.948–5.888)	0.065
*CRY1* and *PER3*	5.800 (1.978–17.004)	0.001

^a^ The reference for the odds ratio is the absence of the corresponding risk factor. CI, confidence interval.
